# Toll-Like Receptor Signaling in Severe Acute Respiratory Syndrome Coronavirus 2-Induced Innate Immune Responses and the Potential Application Value of Toll-Like Receptor Immunomodulators in Patients With Coronavirus Disease 2019

**DOI:** 10.3389/fmicb.2022.948770

**Published:** 2022-06-27

**Authors:** Jiayu Dai, Yibo Wang, Hongrui Wang, Ziyuan Gao, Ying Wang, Mingli Fang, Shuyou Shi, Peng Zhang, Hua Wang, Yingying Su, Ming Yang

**Affiliations:** ^1^Department of Molecular Biology, College of Basic Medical Sciences, Jilin University, Changchun, China; ^2^College of Clinical Medicine, Jilin University, Changchun, China; ^3^Department of Thoracic Surgery, The First Affiliated Hospital of Jilin University, Changchun, China; ^4^Department of Anatomy, College of Basic Medical Sciences, Jilin University, Jilin, China

**Keywords:** SARS-CoV-2, COVID-19, innate immune response, Toll-like receptor, immunomodulator

## Abstract

Toll-like receptors (TLRs) are key sensors that recognize the pathogen-associated molecular patterns (PAMPs) of severe acute respiratory syndrome coronavirus 2 (SARS-CoV-2) to activate innate immune response to clear the invading virus. However, dysregulated immune responses may elicit the overproduction of proinflammatory cytokines and chemokines, resulting in the enhancement of immune-mediated pathology. Therefore, a proper understanding of the interaction between SARS-CoV-2 and TLR-induced immune responses is very important for the development of effective preventive and therapeutic strategies. In this review, we discuss the recognition of SARS-CoV-2 components by TLRs and the downstream signaling pathways that are activated, as well as the dual role of TLRs in regulating antiviral effects and excessive inflammatory responses in patients with coronavirus disease 2019 (COVID-19). In addition, this article describes recent progress in the development of TLR immunomodulators including the agonists and antagonists, as vaccine adjuvants or agents used to treat hyperinflammatory responses during SARS-CoV-2 infection.

## Introduction

Toll-like receptors (TLRs) are members of the pattern recognition receptor (PRR) family, and they play pivotal roles in the activation of innate immune responses and the regulation of cytokine expression (Hedayat et al., 2011; Birra et al., 2020; Debnath et al., 2020). TLRs perform their functions by recognizing distinguishing molecules of invading pathogens, called pathogen-associated molecular patterns (PAMPs), and then initiate innate immune responses *via* several distinct signaling pathways, thereby limiting infection and promoting adaptive immune responses (Kawai and Akira, 2011). Generally, myeloid differentiation primary response 88 (MyD88) and Toll-IL-1R (TIR)-domain containing adaptor-inducing interferon-β (TRIF) are the two main pathways by which TLRs transduce signals after activation (Kim et al., 2018).

The innate immune system acts as the first line of defense against invading pathogens, including the severe acute respiratory syndrome coronavirus 2 (SARS-CoV-2). SARS-CoV-2 is the causative viral pathogen of the coronavirus disease 2019 (COVID-19), and the COVID-19 pandemic began in March 2020 (Tabary et al., 2020). The characteristics of COVID-19 are extremely variable, and the disease ranges from an asymptomatic form lasting for a few days or mild to severe forms of interstitial pneumonia which requires ventilation therapy and can lead to death (Khan et al., 2020). TLRs recognize SARS-CoV-2-derived molecules and activate innate immune responses. Many studies have shown that SARS-CoV-2 activates the innate immune system *via* TLRs, and mediates the upregulation of TLR expression, which contribute to the elimination of the virus (Aboudounya and Heads, 2021; Bortolotti et al., 2021; Sariol and Perlman, 2021). However, TLR activation may act as a double-edged sword, and dysregulated TLR responses may lead to persistent inflammation and tissue destruction (Yokota et al., 2010; Ebermeyer et al., 2021). For instance, the severity of COVID-19 is associated with cytokine storms in patients which could be produced by overactivation of TLR pathways (Tang et al., 2020; Manik and Singh, 2021). Therefore, a better understanding of the relationship between TLRs and SARS-CoV-2 is critical for understanding the immunopathogenesis involved in COVID-19 and for the preventive and therapeutic application of TLR immunomodulators to combat the disease. In this review, we provide an overview of our recent understanding of the roles of TLRs in SARS-CoV-2-induced immune responses, and we discuss the potential prophylactic and/or therapeutic value of TLR agonists or antagonists in COVID-19 patients.

## Structure and Classification of Toll-Like Receptors

All TLRs are type I transmembrane proteins that consist of a leucine-rich repeat (LRR) module, a transmembrane region, and a TIR domain. The LRR motif includes 19–25 tandem sequences containing leucine and is responsible for the recognition of PAMPs, while the TIR endodomain functions to initiate intracellular signaling events *via* downstream adaptors. According to the composition of their subunits, TLRs operate as heterodimers or homodimers. The classification and expression of TLRs differs between species. Thirteen TLRs have been discovered in mice. However, TLR11 has no function, and the TLR12 and TLR13 genes are absent in the human genome. Generally, the TLR family can be divided into two subgroups based on their localization: cell surface TLRs and intracellular TLRs (Kawasaki and Kawai, 2014). Cell surface TLRs reside on the cell membrane and mainly contain TLR1, TLR2, TLR4, TLR5, TLR6, and TLR10, while intracellular TLRs include TLR3, TLR7, TLR8, TLR9, TLR11, TLR12, and TLR13 (Table 1). Intracellular TLRs localize to intracellular compartments, such as the endosomes, endoplasmic reticulum, and lysosomes.

**TABLE 1:** Human and murine TLR classification and their natural ligands.

Classification	TLR name	Gene location	Ligands	References
Cell surface TLRs	TLR1	5C3.1 (mouse)/4p14 (human)	Triacylated lipopeptides, lipoarabinomannan	Yu and Feng, 2018; Su et al., 2019
	TLR2	3E3 (mouse)/4q31.3 (human)	Zymosan, peptidoglycan, lipoteichoic acid, endogenous HSP, HMGB1, gp96	Patidar et al., 2018; Yu and Feng, 2018
	TLR4	4C1 (mouse)/9q33.1 (human)	LPS, endogenous HSP, HMGB1, β-defensin 2	Vaure and Liu, 2014; Patidar et al., 2018
	TLR5	1H5 (mouse)/1q41 (human)	Flagellin	Yang and Yan, 2017
	TLR6	5C3.1 (mouse)/4p14 (human)	Diacylated lipopeptides, zymosan, lipoteichoic acid	Yu and Feng, 2018
	TLR10	4p14 (human)	Unknown	Kawasaki and Kawai, 2014
Intracellular TLRs	TLR3	8B1.1 (mouse)/4q35.1 (human)	dsRNA	Turton et al., 2020
	TLR7	XF5 (mouse)/Xp22.2 (human)	ssRNA	Tanji et al., 2013
	TLR8	XF5 (mouse)/Xp22.2 (human)	ssRNA	Tanji et al., 2013
	TLR9	9F1 (mouse)/3p21.2 (human)	Unmethylated CpG DNA, 5′-xCx DNA	Ohto et al., 2018
	TLR11	11C1 (mouse)	Profilin of *Toxoplasma gondii*	Lester and Li, 2014
	TLR12	4D2.2 (mouse)	Profilin of *Toxoplasma gondii*	Lester and Li, 2014
	TLR13	XD (mouse)	Bacterial 23S rRNA	Oldenburg et al., 2012

Cell surface TLRs mainly recognize microbial membrane components such as lipoproteins, lipids, and proteins. TLR2 forms heterodimers with either TLR1 or TLR6. It recognizes a wide variety of PAMPs including lipoproteins, lipopeptides, peptidoglycans, and zymosan (Yu and Feng, 2018). TLR4 is mainly expressed on cells of the immune system, including macrophages, monocytes, and dendritic cells (DCs). TLR4 recognizes bacterial lipopolysaccharide (LPS) and its activation leads to the synthesis of proinflammatory cytokines and chemokines (Vaure and Liu, 2014). TLR5 is known to specifically sense and recognize bacterial flagellins (Yang and Yan, 2017). The ligands of TLR10 remain unclear but it is believed that human TLR10 collaborates with TLR2 to recognize ligands from *Listeria* and senses influenza A virus during infection; however, TLR10 has no function in mice due to an insertion of a stop codon (Kawasaki and Kawai, 2014).

Intracellular TLRs can recognize nucleic acids derived from foreign pathogens and self-derived nucleic acids in the context of some autoimmune diseases (Schlee and Hartmann, 2016). TLR3 is widely distributed in various epithelial cells, fibroblasts, nerve cells and immune cells. TLR3 mainly recognizes viral double-stranded RNA (dsRNA), self-RNAs derived from damaged cells and the replication intermediates generated during the life cycle of single-stranded RNA (ssRNA) viruses and DNA viruses (Turton et al., 2020). TLR7 is localized to endosomes and is mainly expressed by B cells, monocytes and plasmacytoid DCs (pDCs), while TLR8 expression is closely associated with conventional DCs (cDCs). TLR7/8 can recognize ssRNA and activate MyD88-dependent pathways, which lead to the subsequent production of type I interferons (IFNs) and inflammatory cytokines (Kawai and Akira, 2011; Jung and Lee, 2021). TLR9 recognizes unmethylated CpG-DNA motifs which are frequently presented in viral and bacterial DNA, and activates the innate immune response to eliminate these pathogens. In addition, TLR9 also binds to the DNA sequences that contain cytosine in the second position from the 5′ end (5′-xCx DNA), which cooperatively promotes dimerization and activation of TLR9 in the presence of CpG DNA (Ohto et al., 2018). TLR11 is located to endolysosomes, and TLR12 is predominantly expressed in myeloid cells. These TLRs function in recognizing profilin from the parasites (Lester and Li, 2014). TLR13, the orphan receptor in mice, was found to recognize a conserved 23S ribosomal RNA (rRNA) sequence in bacteria (Wang et al., 2016).

## Toll-Like Receptor Recognition of Severe Acute Respiratory Syndrome Coronavirus 2 Components

### Structure of Severe Acute Respiratory Syndrome Coronavirus 2

Viral proteins and nucleic acids can serve as PAMPs, which are sensed by TLRs to induce the production of antiviral and inflammatory cytokines. Thus, it is quite necessary to understand the nucleic acid composition and protein structure of SARS-CoV-2. Similar to other coronaviruses, SARS-CoV-2 is a positive-sense single-stranded RNA (+ssRNA) virus and its genome is approximately 29.9 kb in size; this genome mainly encodes four major structural proteins, including the spike (S), nucleocapsid (N), membrane (M), and envelope (E) proteins. The S protein consists of the receptor binding subunit S1 and the membrane fusing subunit S2. S1 includes a N-terminal domain (NTD), receptor-binding domain (RBD), subdomain 1 (SD1) and subdomain 2 (SD2). S2 includes a fusion peptide (FP), heptad repeat 1 (HR1), heptad repeat 2 (HR2), and transmembrane (TM). The rest of the genome includes two major open reading frames (ORFs)-ORF1a and ORF1b, which can be translated to pp1a and pp1b polypeptides, and then cleaved to form 16 non-structural proteins (Zhang et al., 2021).

### Toll-Like Receptors Recognize Viral Proteins and RNA of Severe Acute Respiratory Syndrome Coronavirus 2

**FIGURE 1:**
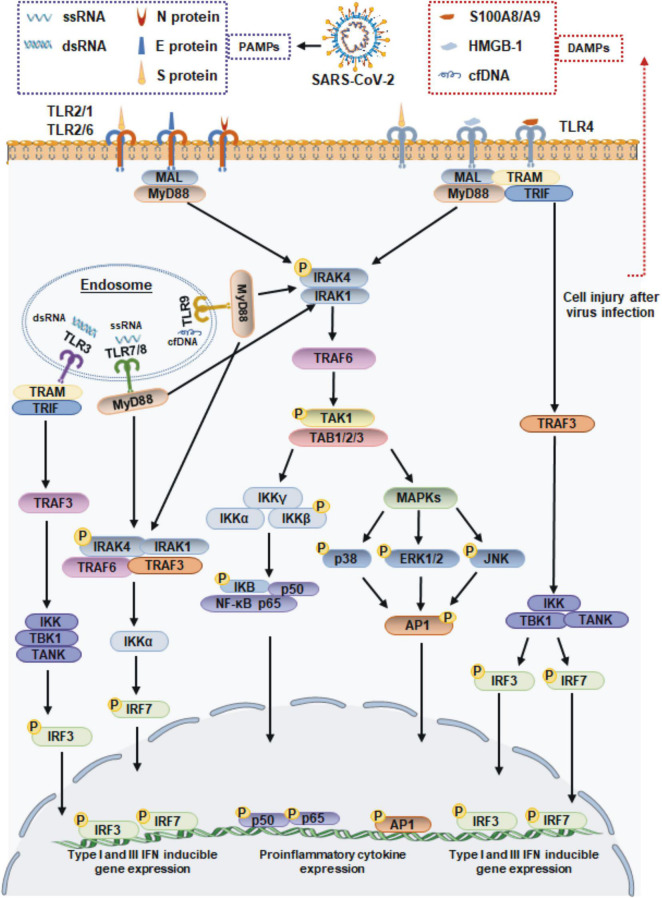
Toll-like receptor-mediated antiviral and inflammatory responses during SARS-CoV-2 infection. TLR1/2/6 and 4 localize to cell membrane, and TLR3, TLR7/8, and 9 localize to endosome surface. The viral proteins of SARS-CoV-2 signal through TLR2 and TLR4 to activate the adaptor MyD88, which subsequently signals *via* NF-κB and MAPK to promote the expression of proinflammatory cytokines. TLR4 also recruits the adapter protein TRIF, which activates the TRAF3, resulting the IRF3 activation to lead the production of type I and III IFN. The ssRNA or dsRNA replication intermediates of SARS-CoV-2 are recognized by TLR3 and TLR7/8, respectively. The TLR7/8 recruit MyD88, and TLR3 *via* TRIF molecule. The proinflammatory cytokines induced by DAMPs accumulating during SARS-CoV-2 infection are driven by the transcription factor NF-κB. MAL, MyD88 adapter-like; TRAF, tumor necrosis factor receptor-associated factor; TRIF, TIR-domain-containing adapter-inducing interferon-β; TRAM, TRIF-related adaptor molecule; IRAK1/4, interleukin-1 receptor associated kinase 1/4; TAK1, transforming growth factor β-activated kinase 1; TAB, TAK1-binding proteins; IKK, IkB kinase; TBK1, TANK-binding kinase 1; ERK1/2, extracellular signal-regulated kinases 1/2; JNK, c-Jun N-terminal kinase; AP-1, activating protein-1.

When an infection occurs, viruses enter the body and adhere to cell surfaces. Cell surface TLRs are most likely to be involved in recognizing molecular patterns from SARS-CoV-2 to induce immune responses. The S protein is a major structural protein and is essential for the interaction of SARS-CoV-2 with host cell receptors. In addition to its direct binding to angiotensin-converting enzyme II (ACE2), the S protein is a potent viral PAMP that is sensed by TLR2 in macrophages, monocytes, and lung epithelial cells. Then TLR2 forms heterodimers with TLR1 or TLR6, promoting formation of a complex containing MyD88 with IRAK kinase family members, activating nuclear factor-κB (NF-κB) and mitogen-activated protein kinase (MAPK) signaling, and ultimately leading to the production of inflammatory cytokines and chemokines (Khan et al., 2021). SARS-CoV-2 infection also increases the expression of TLR2 and other molecules, such as melanoma differentiation-associated gene 5 (MDA5), ACE2 and interferon regulatory factor 3 (IRF3) (Mohanty et al., 2021; Yang et al., 2022), and the expression of TLR2 is positively associated with the severity of COVID-19 (Zheng et al., 2021). In addition to the S protein, the immunogenic properties of other structural proteins have also been investigated. Recent reports have found that TLR2 binds to the SARS-CoV-2 E and N proteins, but only the SARS-CoV-2 N protein induces TLR2 activation, which was not observed with other coronavirus-derived N proteins (Qian et al., 2021; Zheng et al., 2021). TLR4 is well known to recognize bacterial LPS. Actually, TLR4 was found to exhibit a very strong ability to bind to the S protein of SARS-CoV-2 by molecular docking studies (Choudhury and Mukherjee, 2020), and a surface plasmon resonance (SPR) assay confirmed that trimeric SARS-CoV-2 S protein, which is presented on the surface of viral particles, directly binds to TLR4 with a high affinity of ∼300 nM (Zhao et al., 2021). These findings suggest that TLR4 also plays a potential role in the recognition of SARS-CoV-2. This hypothesis has been supported by other evidence demonstrating that the S1 subunit of S protein elicits inflammatory responses *in vitro* and signals through TLR4. The direct exposure of microglia, macrophages, or TLR4 signaling transgenic HEK293 cells to S1 could activate the MyD88-dependent pathway, resulting in the upregulated expression of proinflammatory cytokines (Shirato and Kizaki, 2021; Frank et al., 2022). Moreover, the responses induced by S1 could be blocked by the TLR4-specific inhibitor Resatorvid or TLR4 siRNA (Shirato and Kizaki, 2021; Zhao et al., 2021). Taken together, these findings suggested that the TLR2 and TLR4 can sense SARS-CoV-2 structural proteins, and they may function independently to induce inflammatory processes (Figure 1).

When the SARS-CoV-2 binds to a host cell receptor such as ACE2, it fuses to the host cell membrane and releases its contents into the cell. Viral ssRNA and its replication intermediate dsRNA, are likely to be initially detected by host cells *via* TLR7/8 and TLR3 in endosomes. Previous studies showed that SARS-CoV-2 infection of Calu-3/MRC-5 multicellular spheroids induced the activation of both the TLR3 and TLR7/8 RNA sensor pathways (Bortolotti et al., 2021). Subsequently, TLR7/8 signaling engages the IRF-7 and MyD88-NF-κB signaling pathways, leading to the increased production of type I IFNs and proinflammatory cytokines (Jung and Lee, 2021). In particular, TLR3 signaling relies on the TRIF adaptor protein but not the MyD88 adaptor protein. TRIF interacts with TRAF6 and TRAF3, leading to the activation of IRF3, which results in the release of IFN-α and IFN-β (Kawasaki and Kawai, 2014; Figure 1).

## Toll-Like Receptor-Mediated Antiviral and Inflammatory Responses Against Severe Acute Respiratory Syndrome Coronavirus 2: A Double-Edged Sword

It is well-known that TLR signaling plays a key role in host defense against many pathogens including SARS-CoV-2. However, TLR activation can act as a double-edged sword, and it may activate immune-mediated pathogenesis instead of inducing an immune response that defends against pathogens.

### Toll-Like Receptor Signaling as an Antiviral Mechanism During Severe Acute Respiratory Syndrome Coronavirus 2 Infection

The IFN signaling cascade induced by TLR activation is crucial for controlling SARS-CoV-2 infection. As mentioned above, during SARS-CoV-2 infection, the expression of TLRs, such as TLR3 and TLR7, is elevated (Jung and Lee, 2021), and activated TLR3, TLR4 and TLR7/8 can interact with the signaling adaptor TRIF, inducing the phosphorylation and translocation of IRF3 and IRF7 and the transcription of type I and III IFN genes. Mice lacking the TLR3 gene are prone to SARS-CoV-2 infection and have increased mortality rates (Totura et al., 2015). Additionally, TLR copy numbers also play a role in the production of IFN; for example, the TLR7 and TLR8 genes are located on the X chromosome. Therefore, TLR7/8 copy numbers are higher in women than in men and their biallelic expression leads to the stronger activation of TLR7/8 and more production of type I IFN. These phenomena are correlated with higher resistance to SARS-CoV-2 infection and better prognosis in women (Viveiros et al., 2021). Moreover, loss-of-function variants of TLR7 have recently been reported to underlie a strong predisposition to severe COVID-19 in a small number of males (Fallerini et al., 2021). Both type I and III IFNs can induce an antiviral transcriptional program (Park and Iwasaki, 2020), thus, dysregulation of host IFN responses has been shown to be associated with severe disease progression in COVID-19 patients. For example, pDCs use TLR7 to sense ssRNA fragments from viruses that are rich in guanine and uracil (GU rich), and TLR7 seems to mainly act *via* the MyD88-TRAF3/6 pathway, inducing the production of type I IFN, IFN-γ, and IFN-λ3 and the upregulation of CD86 expression (Bortolotti et al., 2021; Salvi et al., 2021). However, the pDCs response of COVID-19-infected patients is functionally impaired (Choi and Shin, 2021).

In addition, peripheral blood immune cells from severe COVID-19 patients exhibit diminished type I and III IFN responses but enhanced proinflammatory IL-6 and TNF-α responses (Blanco-Melo et al., 2020; Hadjadj et al., 2020). Analysis of the serum from critical COVID-19 patients showed the levels of proinflammatory cytokines and chemokines are strongly elevated while the levels of type I and III IFNs are undetectable (Park and Iwasaki, 2020). Notably, SARS-CoV-2 also possesses some strategies to escape the innate immune response due to a wide range of viral proteins, such as the M, NSP6, NSP13, NSP15, ORF3b, ORF6, ORF8, and ORF9b proteins, that affect the IFN-mediated antiviral responses (Jiang et al., 2020; Konno et al., 2020; Lei et al., 2020; Han et al., 2021; Sui L. et al., 2021). Therefore, future investigations are still required to obtain a clear understanding of the inhibition of TLR signaling by SARS-CoV-2.

### Toll-Like Receptor Signaling as a Part of the Inflammatory Response During Severe Acute Respiratory Syndrome Coronavirus 2 Infection

The activation of TLRs plays a dual role in the progression of COVID-19. It is thought that the production of proinflammatory cytokines is induced by the activation of different TLRs, such as TLR2, TLR4, and TLR7/8, which greatly contributes to the pathogenesis of COVID-19 and its severity. Immunopathological processes that cause death in COVID-19 patients occur due to the interaction of TLRs with virus particles (Patra et al., 2021). TLR2 and TLR4 sense viral proteins, and the expression levels of molecules related to the TLR2- and TLR4-inflammatory signaling molecules are upregulated in in COVID-19 patients, which suggest the involvement of TLR2 and TLR4 signaling in the induction of pathological inflammation during COVID-19 (Sohn et al., 2020). Moreover, several studies have shown that an overwhelming TLR7 response may promote the development of severe COVID-19 (Fallerini et al., 2021; Kayesh et al., 2021), highlighting the clinical importance of the TLR-mediated immune response during SARS-CoV-2 infection. Moreover, other studies on SARS-CoV-2 have revealed the pathological role of TLR4 and TLR7/8 in excessive inflammatory response in COVID-19 patients as it leads to the formation of neutrophil extracellular traps (NETs) and the activation of inflammasomes, leading to acute lung injury (Khadke et al., 2020; Sohn et al., 2020; Veras et al., 2020).

Additionally, obese and overweight individuals exhibit significantly increased TLR expression, which impacts the severity of COVID-19. It is hypothesized that desensitization of TLR signaling may occur due to chronic stimulation in obese and elderly people. TLR/MyD88 signaling, which is enhanced in obese individuals, may contribute to the excessive inflammatory response observed during severe infection with SARS-CoV-2 (Cuevas et al., 2021). Similarly, significantly elevated expression of IL-6 and TNF-α was observed to be associated with TLR expression in obese individuals but not in controls (Kayesh et al., 2021). Moreover, delayed IFN responses fail to control the virus and can cause inflammation and tissue damage. Delayed but considerable type I IFN responses in SARS-CoV-2 infected BALB/c mice trigger the accumulation of monocytes and macrophages as well as the production of proinflammatory cytokines, resulting in lethal pneumonia, vascular leakage, and insufficient T-cell responses (Channappanavar et al., 2016).

Furthermore, damage to host cell caused by SARS-CoV-2 infection can also lead to the release of endogenous danger-associated molecular patterns (DAMPs). These endogenous self-antigens, such as high-mobility group box 1 (HMGB1) and heat shock proteins (HSPs), activate MAPK and NF-κB signaling, which triggers an inflammatory response. In COVID-19 patients, HMGB1 can be released from dying cells and functions as a pro-inflammatory inducer to bind with TLR4, leading to the production of IL-1β, IL-6, and TNF-α (Yang et al., 2015; Cicco et al., 2020). Another DAMP shown to regulate inflammation during SARS-CoV-2 infection is the S100A8/A9 complex, which is released from host neutrophils and has been proposed to be a potential biomarker in COVID-19 patients. Mechanistically, the S100A8/A9 complex is also an endogenous ligand of TLR4 on DCs and mediates host proinflammatory responses (Mellett and Khader, 2022). TLR9 is not directly involved in recognition of SARS-CoV-2. However, cell-free DNA (cfDNA) production that is triggered by tissue injury during virus infection, cfDNA can subsequently act as a DAMP and exacerbates inflammation *via* TLR9. Recent studies have reported that elevated levels of cfDNA in COVID-19 patients are strongly correlated with COVID-19 disease severity (Andargie et al., 2021; Cavalier et al., 2021). Moreover, the increased cfDNA levels in COVID-19 patients generates excessive mitochondrial ROS (mtROS) production in a concentration-dependent manner (Andargie et al., 2021). Taken together, these findings suggest that host molecules that are released during SARS-CoV-2 infection may function independently as DAMPs, resulting in a more severe inflammatory response (Figure 1).

## Toll-Like Receptor Agonists as Adjuvants for Coronavirus Disease 2019 Vaccines

More efficient and safe vaccines are still a critical need for combating COVID-19. In vaccine development, adjuvants are required to increase antigen recognition and enhance the magnitude and durability of the elicited immune responses. TLR agonists are considered important molecules for triggering and enhancing rapid and long-term innate immune responses, and they have been extensively studied for use as vaccine adjuvants against cancer and microbial infections. Compared to the first-generation adjuvants, such as aluminum adjuvant, TLR agonist adjuvants could guide DC maturation to elicit a stronger T-cell response. Various TLR agonists, including Pam3CSK4, poly(I:C), monophosphoryl lipid A (MPLA), resiquimod (R848), and CpG oligonucleotide (ODN), are currently under investigation for use as vaccine adjuvants to prevent SARS-CoV-2 infection. The TLR agonists currently under development for COVID-19 vaccines are listed in Table 2.

**TABLE 2:** Toll-like receptor agonists as vaccine adjuvants in COVID-19 vaccine formulation.

TLR agonists	Platform	Adjuvant	Antigen	Formulation	Immunological response			Route	Animal model or clinical trial	References
					Nab	sIgA	T-cell response			
TLR 1/2	Peptide vaccine	XS15	T-cell epitopes from viral protein	Montanide ISA 51 VG	Weak	NA	CD4^+^ T and IFN-γ response	SC	Phase II trial	Rammensee et al., 2021; Heitmann et al., 2022
TLR 1/2 and TLR3	Subunit vaccine	L-pampo	RBD and S1 antigens	NA	Strong	NA	IFN-γ response	IM	BALB/c and ferret	Jeong et al., 2021
TLR3	Adenovirus-based vaccine	dsRNA	SARS-CoV-2 S and N gene	Adenovirus	Strong	Moderate	NA	Oral	Hamsters and phase I trial	Tiboni et al., 2021
TLR3	Subunit vaccine	PIKA	Trimeric S antigen	NA	Strong	NA	Balanced Th1/Th2 and IFN-γ response	IM	Rabbits, mice, and non-human primates	Liu Y. et al., 2021
TLR3 and TLR9	Subunit vaccine	CpG ODN + poly I:C + IL-15	S1 protein	PLGA or DOTAP	Weak	Strong	CD4^+^ T response	IM, IN	Rhesus macaques	Sui Y. et al., 2021
TLR4	Subunit vaccine	MPLA + PUUC	S1 protein	Polymer nanoparticles	Strong	Strong	Memory T-cell response	IN, IM	BALB/c	Atalis et al., 2022
TLR-4	Subunit vaccine	MPLA + GM-CSF	SARS-CoV-2 N/S1/S2 proteins	MSRs	Strong	NA	CD4^+^ and CD8^+^ T response	SC	BALB/c	Langellotto et al., 2021
TLR7/8	Subunit vaccine	Alhydroxiquim-II	Trimeric spike antigen	NA	Strong	NA	CD4^+^ T	IM	C57BL/6, rabbits, horses	Counoupas et al., 2022
TLR7/8	Subunit vaccine	R848	S1 protein	Nanoparticle decorated erythrocytes	Strong	NA	CD4^+^ T response	IV	C57BL/6	Wang et al., 2021
TLR7/8	Inactivated vaccine	Chemisorbed Algel	Inactivated antigen	NA	Strong	NA	CD4^+^ T and Th1-biased responses	IM	Mice, rats, and rabbits	Ganneru et al., 2021
TLR-7 or TLR-9	Subunit vaccine	AS37-Alum or CpG 1018-Alum	RBD antigen	Self-assembling protein nanoparticle	Strong	NA	CD4^+^ T response	IM	Rhesus macaques and phase II trial	Arunachalam et al., 2021; Richmond et al., 2021
TLR9	mRNA vaccine	CpG SD-101	RBD mRNA	CART	Strong	NA	CD4^+^ and CD8^+^ T	IV, IM	BALB/c	Haabeth et al., 2021
TLR-9	Subunit vaccine	CpG 7909-Alum	Trimeric S antigen	NA	Strong	NA	CD4^+^ T response	IM	BALB/c and monkeys	Liu H. et al., 2021

*NA, not available; Nab, naturalization antibody; SC, subcutaneous; IM, intramuscular; IV, intravenous; IN, intranasal; PUUC, RIG-I agonist; PLGA, poly(lactic-co-glycolic acid); DOTAP, 1,2-dioleoyl-3-trimethylammonium-propane; CART, charge-altering releasable transporters.*

### Toll-Like Receptor 1/2 Agonists

The TLR1/TLR2 ligand Pam3CSK4 is a synthetic triacylated lipopeptide and can activate the proinflammatory transcription factor NF-κB. A new water-soluble synthetic Pam3CSK4-derivative, named XS15, is being used in combination with peptides from the SARS-CoV-2 spike protein to develop a vaccine format that induces CD4^+^ T-cell responses against peptides predicted to bind to HLA-DR (Rammensee et al., 2021). In a phase I open-label trial, a peptide-based vaccine CoVac-1, which is composed of T-cell epitopes derived from various SARS-CoV-2 proteins, was combined with XS15 and emulsified in Montanide ISA 51 VG. This vaccine induced profound SARS-CoV-2-specific T-cell responses targeting multiple vaccine peptides in all the study participants. Moreover, the interferon (IFN)-γ T-cell responses induced by CoVac-1 persisted in the follow-up analyses and surpassed those detected after vaccination with approved vaccines (Heitmann et al., 2022). In addition, in a study of a SARS-CoV-2 subunit vaccine, a combination of TLR1/2 and TLR3 agonists (L-pampo) was found to be a potent adjuvant for eliciting a neutralization antibody response and an antigen-specific cellular immune response against SARS-CoV-2, resulting in a substantially decreased viral load in a ferret model (Jeong et al., 2021).

### Toll-Like Receptor 4 Agonists

The TLR4 ligand LPS can regulate inflammation and effector T-cell differentiation. MPLA is a modified form of LPS that exhibits strong immune stimulatory activity but avoids most inflammatory toxicity. It has been approved for utilization as an adjuvant in vaccines against human papilloma virus and hepatitis B virus. In a previous vaccine study, MPLA-adjuvanted truncated spike protein fused with Fc of human IgG (S377-588-Fc) induced a significantly higher titer of specific IgG antibodies against Middle East respiratory syndrome coronavirus than did the alum-adjuvanted protein. In particular, MPLA-adjuvanted S377-588-Fc protein elicited a stronger Th2 (IgG1)-biased response (Zhang et al., 2016). Recently, a biomaterial COVID-19 vaccine based on mesoporous silica rods (MSRs) and loaded with MPLA, granulocyte-macrophage colony-stimulating factor (GM-CSF), and SARS-CoV-2 viral protein antigens was shown to slowly release their cargo and form subcutaneous scaffolds that recruited and activated antigen-presenting cells (APCs) at the local site to generate adaptive immune responses (Langellotto et al., 2021). The SARS-CoV-2 is still mutating; however, the MPLA-adjuvanted antigens like S-trimer/MPLA, RBD/MPLA, and S1/MPLA remain to induce a strong humoral and cellular immune responses against spike variants, including alpha, beta, gamma, delta, and omicron (Wang et al., 2022). Another TLR4 agonist, inulin acetate (InAc), which is a plant-based polymer, has been reported to induce high IgG1, IgG2a, and sIgA titers against antigens in serum after intranasal immunization using antigen-loaded InAc nanoparticles (InAc-NPs); this approach resulted in a strong memory response indicative of both humoral and cellular immune activation, and may be useful in the development of a COVID-19 vaccine (Bakkari et al., 2021).

### Toll-Like Receptor 7/8 Agonists

Toll-Like Receptor 7/8 activation may be investigated as an additional strategy for anti-SARS-CoV-2 vaccines address the current challenge of viral escape. TLR7 stimulation may help viral clearance through Th1 antiviral responses as well as exert beneficial broncho-vasodilatory activity (Khalifa and Ghoneim, 2021). As a synthetic and selective ligand for TLR7, imiquimod has been approved for human-papillomavirus-induced genital and perianal warts. An imiquimod analog, resiquimod (R848), was proven to be a dual TLR7 and TLR8 synthetic agonist that elicits prominent IFN-α/β and IL-6 responses and robust cytotoxic T-cell (CTL) and B-cell proliferation; thus, it is particularly suited for antiviral immune responses. Indeed, the cytokine profiles induced by R848, are almost identical to the profiles induced by the licensed mRNA vaccines against COVID-19 (Rossmann et al., 2021). In a SARS-CoV-2 virus-mimetic nanoparticle vaccine, the SARS-CoV-2 spike protein S1 subunit and R848 were attached to erythrocytes and injected into mice, resulting in greater maturation and activation of APCs, production of specific IgG antibodies, and systemic antiviral T-cell responses than the nanoparticles alone (Wang et al., 2021). However, R848 adjuvanticity should stress more on vaccine formulation. A recent study showed that R848 conjugated to multilamellar liposomes rather than forming a linear structure, resulting in stronger immunostimulatory activity (Liang et al., 2020). Moreover, an R848-encapsulating poly lactic-co-glycolic acid (PLGA) nanoparticle can reduce the excessive level of inflammatory cytokines induced by free R848 (Chen et al., 2021), which could be beneficial for providing an appropriate immune response and long-term safety in vaccine development.

### Toll-Like Receptor 3/9 Agonists

Optimal protection against coronavirus probably involves neutralizing antibodies and CD8^+^ T cells. Among the TLR agonists, the TLR3 and TLR9 ligands, poly(I:C) and CpG ODN significantly augment the CD8^+^ T-cell responses to a greater extent than other adjuvants. Thus, CpG ODN and poly(I:C) have been utilized as adjuvants in influenza vaccines. Studies have demonstrated that CpG ODN or PIKA [a stabilized derivative of poly(I:C)] can stimulate enhanced IgG production in animals have been used in the context of immunization with an inactivated SARS-CoV-2 vaccine (Gai et al., 2008; Gupta and Gupta, 2020). Moreover, compared with the alum adjuvanted SARS-CoV-2 S1 vaccine, intranasal boosting with nanoparticles, formulating with CpG 1018 and poly(I:C), elicits higher dimeric IgA production, IFN-α production, and T-cell activation in rhesus macaques (Sui Y. et al., 2021). In another study, a recombinant S trimeric protein adjuvanted with PIKA was reported to induce high titers of SARS-CoV-2 neutralizing antibodies and to protect non-human primates from virus challenge (Liu Y. et al., 2021). Another CpG ODN 7909-adjuvanted SARS-CoV-2 vaccine, 202-COV, is a S-protein subunit vaccine formulated with aluminum hydroxide; that elicits robust neutralizing antibody responses and substantial CD4^+^ T-cell responses in both mice and non-human primates; this vaccine is currently being investigated in phase II studies of COVID-19 (Liu H. et al., 2021). CpG ODN is capable of inducing both cellular and humoral immune responses, and it preferentially induces Th1-biased responses. Mice that were immunized with mRNA encoding the spike protein of SARS-CoV-2, co-formulated with CpG ODN, developed therapeutically relevant levels of RBD-specific neutralizing antibodies in both circulation and lung bronchial fluids. In addition, vaccination elicited strong and long-lasting RBD-specific Th1 T-cell responses including CD4^+^ and CD8^+^ T-cell memory responses (Haabeth et al., 2021).

## Toll-Like Receptor Signaling Inhibitors Protect Against Hyperinflammatory Response in Severe Acute Respiratory Syndrome Coronavirus 2 Infection

Uncontrolled TLR-mediated inflammation has been suggested to contribute to immunopathological consequences in COVID-19 patients. It is quite obvious that targeted manipulation of TLR signaling decreases excessive inflammatory responses. TLR4 is one of the major contributors to SARS-CoV-2 infectivity and pathogenesis, and its antagonists are capable of inhibiting the harmful effects of TLR4 signaling. For example, Lipid X is a Lipid A biosynthetic precursor that can competitively bind to TLR4 to block cytokine production *via* the downstream signaling pathways. Therefore, many TLR4 modulators, both natural and synthetic, can be investigated in the context of COVID-19 treatment. TAK242 is a potent and selective TLR4 antagonist that inhibits the release of inflammatory cytokines from human THP-1 cells after exposure to SARS-CoV-2. Therefore, it is postulated that TAK242 may improve patient outcomes by dampening the inflammatory response and preventing systemic infection in patients with COVID-19 (Kate Gadanec et al., 2021).

The intracellular RNA sensors TLR3 and TLR7/8 are thought to be involved in the hyperinflammatory responses induced by SARS-CoV-2 infection. A TLR7/8 antagonist, Enpatoran (M5049), is a potent dual TLR7/8 inhibitor that is expected to cease the hyperinflammatory milieu in symptomatic patients with COVID-19. Furthermore, Merck has already initiated a phase II randomized, controlled clinical study evaluating the efficacy and safety of M5049 in the COVID-19 patient population (Khalifa and Ghoneim, 2021; Port et al., 2021). Similarly, famotidine, a specific histamine H2 receptor antagonist, can inhibit TLR3 expression in SARS-CoV-2 infected cells and reduce TLR3-dependent NF-κB and IRF3 signaling, subsequently controlling antiviral and inflammatory responses (Mukherjee et al., 2021) and reducing the risk of intubation and death in hospitalized patients with COVID-19 (Freedberg et al., 2020). Another TLR signaling inhibitor, the PPARα agonist oleoylethanolamide (OEA), was reported to attenuate TLR3-induced hyperthermia and reduce the expression of hyperthermia-related genes including IL-1β, iNOS, COX2, and m-PGES in the hypothalamus (Flannery et al., 2021).

Toll-like receptor antagonists are not only used as individual drugs but may also be used in combination with immunomodulatory drugs in severe cases of COVID-19 to enhance potential synergistic effects and possibly reduce adverse effects. Currently, most TLR antagonists are being investigated in clinical trials to evaluate their efficacy in reducing detrimental immune effects without causing a drastic change in their basal levels to maintain the immune homeostasis.

## Conclusion

Toll-like receptors are important constituents of the innate immune system and can recognize a wide variety of PAMPs from viruses. This review has described the various TLRs that are involved in the immunopathogenesis of SARS-CoV-2 infection and the effects of application of TLR immunomodulators in patients with COVID-19. Of the TLRs that have been identified, TLR7/TLR8 and TLR3 are intracellular receptor that sense viral ssRNA, and dsRNA replication intermediates, respectively. TLR2 and TLR4 reside on the cell surface and are activated by SARS-CoV-2 glycoproteins. TLR signaling elicits antiviral and proinflammatory cytokine production through MyD88-dependent and/or TRIF-dependent pathways. The activation of the innate immune response often contributes to viral clearance and disease resolution. Therefore, TLR agonists could be formulated as adjuvants for use with the S protein or RBD to enhance neutralizing antibody production and T-cell responses against SARS-CoV-2 infection since they elicit timely and optimal TLR responses. However, dysregulated immune signaling may lead to the detrimental production of proinflammatory cytokines and chemokines that cause severe disease. Thus, the use of TLR antagonists might exert a beneficial effect, by attenuating deleterious hyperinflammatory responses in severe COVID-19 patients. However, most of these new therapeutics or combination strategies for anti-SARS-CoV-2 infection are currently being studied in clinical trials of various phases.

## Author Contributions

JD, YBW, and HRW contributed to the writing of the manuscript. MY and YS provided the ideas and revised the draft manuscript. ZG, YW, MF, SS, PZ, and HW approved the version to be published. MF provided a professional English language revision. All authors read and approved the final manuscript.

## Conflict of Interest

The authors declare that the research was conducted in the absence of any commercial or financial relationships that could be construed as a potential conflict of interest.

## Publisher’s Note

All claims expressed in this article are solely those of the authors and do not necessarily represent those of their affiliated organizations, or those of the publisher, the editors and the reviewers. Any product that may be evaluated in this article, or claim that may be made by its manufacturer, is not guaranteed or endorsed by the publisher.
